# Parks and Health: Aligning Incentives to Create Innovations in Chronic Disease Prevention

**DOI:** 10.5888/pcd11.130407

**Published:** 2014-04-17

**Authors:** Meredith A. Barrett, Daphne Miller, Howard Frumkin

**Affiliations:** Author Affiliations: Daphne Miller, University of California San Francisco, San Francisco, California; Howard Frumkin, University of Washington School of Public Health, Seattle, Washington.

Federal, state, and local park agencies across the nation have faced budget cuts in recent years as a result of the economic downturn and sequestration, resulting in park closures, reduced hours, staffing cuts, and deferred maintenance. For example, California faced closure of 70 state parks in 2012 to trim $22 million from the strapped state budget. Although most parks were saved by contributions from various partners, future funding remains tenuous. Perhaps the initial proposal to shutter 25% of California’s state parks to save 0.14% of the state budget deficit would not have been made if we valued these lands not only for recreation and conservation but also as a critical public health resource. With health care reform shifting incentives toward prevention, now is the time to look for low-cost, high-yield wellness opportunities, such as those offered by parks and other green spaces.

Parks are appealing venues for physical activity. Obesity and sedentary lifestyles are linked to a host of chronic diseases, including diabetes, heart disease, cancer, hypertension, arthritis, stroke, depression, and sleep disorders, which account for more than 20% of total US health care costs (1). Projections indicate that millions of Americans will be newly diagnosed with a preventable chronic disease over the next 20 years at an estimated cost ranging from $48 billion to $66 billion per year (2). Physical activity is a proven strategy to prevent, manage, and reduce this burden, but only an estimated 50% of the US population gets enough exercise (3). Access to outdoor space is associated with initiating and maintaining physical activity and reducing obesity, especially when that space is well maintained, safe, and accessible and offers attractive facilities and programs.

In addition to physical activity benefits, parks may promote mental health, social cohesion, and general well-being (4). (Please also see the supplemental bibliography in the Appendix.) As far back as the mid-19th century, Fredrick Law Olmsted, widely considered one of the founders of landscape architecture and the American Parks Movement, believed that naturalistically designed parks could counter the stress of urban living by offering a transformative environmental experience that enabled people to regain both mental and physical health (5). Olmstead believed intuitively that polluted city air could be “disinfected by sunlight and foliage.” He also designed parks with societal inclusion in mind as a place for members of all socioeconomic strata to enjoy and build community through shared aesthetic experience. Interestingly, his son, Frederick Law Olmsted Jr., conducted the initial survey of potential state park land in California and guided the nascent state park commission in the purchase of its first properties in 1928.

Mounting scientific evidence corroborates Olmsted’s intuition. Physical activity in natural park settings may confer benefits above and beyond equivalent activity in built settings (6). Proximity to parks and green space has been associated with reductions in self-reported stress, depressive symptoms, and interpersonal violence and with improved attention, self-discipline, social ties, and quality of life (4).

Access to parks and green space may also help reduce health disparities. Populations with low socioeconomic status in urban settings are likely to be deprived of such access, and contact with nature has the potential to disproportionately improve health outcomes for these populations (7).

To tap into the potential of parks and green space, nature–health collaborations are gaining popularity nationwide. Health facilities around the country are adopting “park prescriptions”; insurers and health plans are sponsoring trails and parks and encouraging subscribers to use them. Highly publicized programs such as Michelle Obama’s “Let’s Move Outside” campaign, the “Leave No Child Inside” campaign, the Children and Nature Network, and the National Park Service’s “Healthy Parks, Healthy People” initiative have sparked a conversation about nature and green space as a health resource.

Despite a rich history and the renewed interest in the health benefits of green space, our public lands continue to be an undervalued and unequally used health resource. In California, for example, visitors made an impressive 65 million trips to state parks last year (1.7 visits per capita), generating $4 billion in economic activity and burning more than 8.9 billion calories (calculated for 30 minutes of moderate physical activity based on the Centers for Disease Control and Prevention estimate of 280 calories expended per hour) (8). Collectively, the nation’s state parks attract more than 720 million visits annually; the National Park Service reported more than 282 million visits in 2012, and 85% of local park directors reported increasing use in a Resources for the Future survey (9–11). Although adequate data exist for absolute visitation rates, more nuanced data are limited on type of use, duration of use, and segmentation of users by demographic and health status. These data are needed because many of these visits represent repeat events from a subpopulation of frequent users, which means that only a segment of the population receives the health benefits of parks.

Many people, because of lack of access, transportation, or familiarity, visit parks infrequently or not at all (9). Physical activity and frequency of park use depend on demographic, socioeconomic, and regional characteristics (12) and reflect disparities in park distribution. Studies have shown that fewer public resources, such as parks, trails, and playgrounds, exist in communities with lower and medium socioeconomic status (12,13) than in communities with higher socioeconomic status. This lack of facilities is associated with decreased physical activity and increased overweight (13), and lack of green space is associated with higher risk for all-cause mortality, an effect that is especially marked for low-income populations (7).

Improving distribution, access, and use of parks — from large national and state parks to small, nearby neighborhood parks — could yield a range of health benefits. For instance, enhanced park use and physical activity sufficient to achieve just a 5% reduction in the burden of diabetes, hypertension, and related conditions could save an estimated $24.7 billion annually in avoided health care costs (1). Adults who satisfied the recommended level of activity (150 minutes of moderate-intensity activity per week) demonstrated a 40% lower relative risk for cardiovascular disease mortality (3). Enhanced park use and activity could also have beneficial effects on mental health, such as reduced tension, depression, and anger and increased social cohesion and its attendant health benefits (6). Economic and environmental benefits of parks outside the health sector, such as increased property values and property tax revenues, storm water management, carbon sequestration, and urban heat island mitigation, would add to overall benefits.

To achieve these gains, municipalities must also address the actual and perceived risks of increased park use, such as safety concerns (9,12) (eg, crime, vandalism, graffiti, lost persons, inadequately maintained equipment), the risk of injury during physical activity, overexertion (eg, cardiac events, heat stroke, dehydration), allergic reactions (eg, poison ivy and oak, bees), and other concerns, such as increased local traffic. The benefits of physical activity far exceed the risk of musculoskeletal injury or sudden cardiac mortality caused by moderate-intensity activity (3).

Creating linkages between health services and parks and building green opportunities into every state and local health prevention strategy is a win–win strategy to promote environmental sustainability and community health. Thinking of our public land as a public health resource offers the potential to develop innovative operating models such as partnerships with health-related business concerns. Could outdoor gear companies share in the cost and revenue of park operations? Could large health insurance payers make financial contributions to parks because of their interest in community-level disease prevention? Could large companies with a demonstrated commitment to employee wellness support park programs to optimize their employees’ health, happiness, and productivity? The opportunity and need exist for communities to become more innovative in park offerings, and partnerships are already forming across the United States (9,12). Parks could position themselves as “green health spaces” and locations for wellness activities and information. This would provide an opportunity for local university students to serve as physical activity ambassadors in parks, much like the successful Health Leads initiative, in which student advocates embedded in clinical settings connect patients with wellness resources (14).

At a time when we need creative “upstream” solutions to reduce health care costs and improve the health and resilience of the US population, it seems counterproductive to neglect a widely distributed and affordable health resource, our public green spaces. Instead of park closures, now is the time for park enhancement and the building of partnerships across the park and health sectors. Indeed, the numbers show that an ounce of green prevention could result in many pounds of cure.

## Appendix: Supplemental Bibliography

This file is available for download as a Microsoft Word document.
