# Supplementary material for: Parks and Health: Aligning Incentives to Create Innovations in Chronic Disease Prevention
Source: Prev Chronic Dis. 2014 Apr 17;11:E63. doi: 10.5888/pcd11.130407 (PMC3993093; doi:10.5888/pcd11.130407)
Supplement: Supplementary file 1 [file 13_0407_01.doc]

**APPENDIX**

**Supplemental Bibliography**

**Parks and Health: Aligning Incentives to Create Innovations in Chronic Disease Prevention**

Alcock I, White MP, Wheeler BW, Fleming LE, Depledge MH. Longitudinal effects on mental health of moving to greener and less green urban areas. Environ Sci Technol 2014; 48: 1247-55.

American Academy of Pediatrics, Committee on Environmental Health. The built environment: designing communities to promote physical activity in children. Pediatr 2009;123:1591-8.

Barton J, Pretty J. What is the best dose of nature and green exercise for improving mental health? a multi-study analysis. Environ Sci Technol 2010;44:3947–3955.

Bedimo-Rung AL, Mowen AJ, Cohen DA. The significance of parks to physical activity and public health: a conceptual model. Am J Prev Med 2005;28(2):159-68.

Bell JF, Wilson JS, Liu GC. Neighborhood greenness and 2-year changes in body mass index of children and youth. Am J Prev Med 2008;35(6):547-53.

Berman MG, Kross E, Krpan KM, Askren MK, Burson A, Deldin PJ, et al. Interacting with nature improves cognition and affect for individuals with depression. J Affect Disord 2012;140:300-05.

Beyond crisis: recapturing excellence in California’s State Park system. Sacramento (CA): Little Hoover Commission; 2013. <http://www.lhc.ca.gov/studies/215/report215.html>. Accessed July 7, 2013.

Children and Nature Network natural leaders. Minneapolis (MN): Children and Nature Network; 2014. <http://www.childrenandnature.org/>. Accessed January 21, 2014.

de Vries S, van Dillen SME, Groenewegen PP, Spreeuwenberg P. Streetscape greenery and health: stress, social cohesion and physical activity as mediators. Soc Sci Med 2013;94:26-33.

Dietary guidelines for Americans. Washington (DC): US Department of Agriculture and US Department of Health and Human Services; 2005. http://www.health.gov/dietaryguidelines/dga2005/document/pdf/DGA2005.pdf. Accessed January 7, 2014.

Ela, GK. Epidemiology of wilderness search and rescue in New Hampshire, 1999–2001. Wilderness Environ Med 2004; 15(1):11-17.

Faber Taylor A, Kuo FE. Children with attention deficits concentrate better after walk in the park. J Atten Disord 2009;12:402-09.

Faber Taylor A, Kuo FE, Sullivan SC. Views of nature and self-discipline: evidence from inner city children. J Environ Psychol 2002;22:49-63.

Floyd MF, Bocarro JN, Smith WR, Baran PK, Moore RC, Cosco NG, et al. Park-based physical activity among children and adolescents. Am J Prev Med 2011;41(3):258-65.

Giles-Corti B, Broomhall MH, Knuiman M, Collins C, Douglas K, Ng K, et al. Increasing walking: how important is distance to, attractiveness, and size of public open space? Am J Prev Med 2005;28(2):169-76.

Gordon-Larsen P, Nelson MC, Page P, Popkin BM. Inequality in the built environment underlies key health disparities in physical activity and obesity. Pediatr 2006;117:417-24.

Kuo FE, Sullivan WC. Environment and crime in the inner city: does vegetation reduce crime? Environ Behav 2001;33(3):343–67.

Kuo FE, Sullivan WC, Coley RL, Brunson L. Fertile ground for community: inner-city neighborhood common spaces. Am J Commun Psychol 1998;26(6):823-851.

Leave No Child Inside. Columbus (OH): Ohio Leave No Child Inside Collaboratives; 2013. <http://www.ohiolnci.org/>. Accessed December 23, 2013.

Let’s Move Outside. Washington (DC): Let’s Move; 2013. <http://www.letsmove.gov/lets-move-outside>. Accessed December 23, 2013.

Louv R. The nature principle: reconnecting with life in a virtual age. Chapel Hill (NC): Algonquin Books; 2012.

McCurdy L, Winterbottom K, Mehta S, Roberts J. Using nature and outdoor activity to improve children's health. Curr Probl Pediatr Adol Hlth Care 2010;40(5):101-118.

Miles R, Coutts C, Mohamadi A. Neighborhood urban form, social environment, and depression. J Urban Health 2011;89:1-18.

Nielsen TS, Hansen KB. Do green areas affect health? Results from a Danish survey on the use of green areas and health indicators. Health Place 2007;13(4):839-50.

Park prescriptions. Ashburn (VA): National Recreation and Park Association; 2013. <http://www.nrpa.org/Grants-and-Partners/Recreation-and-Health/Park-Prescriptions/>. Accessed December 23, 2013.

Potwarka LR, Kaczynski AT, Flack AL. Places to play: association of park space and facilities with healthy weight status among children. J Commun Health 2008;33:344-50.

Preventing chronic diseases: investing wisely in health. Atlanta (GA): Centers for Disease Control and Prevention; 2008. <http://www.cdc.gov/nccdphp/publications/factsheets/prevention/pdf/obesity.pdf>. Accessed January 18, 2014.

Roemmich JN, Epstein LH, Raja S, Yin L, Robinson J, Winiewicz D. Association of access to parks and recreational facilities with the physical activity of young children. Prev Med 2006;43(6):437– 41.

Stephens BD, Diekema DS, Klein EJ. Recreational injuries in Washington state national parks. Wilderness Environ Med 2005; 16(4): 192-197.

Stigsdotter UK, Ekholm O, Schipperijn J, Toftager M, Kamper-Jørgensen F, Randrup TB. Health promoting outdoor environments: associations between green space, and health, health-related quality of life and stress based on a Danish national representative survey. Scand J Public Health 2010; 38: 411-17.

The health and social benefits of recreation. Sacramento (CA): California State Parks; 2005. [http://www.parks.ca.gov/pages/795/files/benefits%20final%20online%20v6-1-05.pdf](http://www.parks.ca.gov/pages/795/files/benefits final online v6-1-05.pdf). Accessed December 27, 2013.

What is Healthy Parks, Healthy People? Washington (DC): National Park Service; 2013. <http://www.nps.gov/public_health/hp/hphp.htm>. Accessed December 23, 2013.

2008 Physical activity guidelines for Americans. Washington (DC): US Department of Health and Human Services; 2008. <http://www.health.gov/paguidelines/guidelines/>. Accessed January 7, 2014.
